# *Codonoboea* (Gesneriaceae) in Terengganu, Peninsular Malaysia, including three new species

**DOI:** 10.3897/phytokeys.131.35944

**Published:** 2019-09-02

**Authors:** Ruth Kiew, Chung-Lu Lim

**Affiliations:** 1 Forest Research Institute Malaysia, 52109 Kepong, Selangor, Malaysia Forest Research Institute Malaysia Kepong Malaysia

**Keywords:** Checklist, key, new species, *Codonoboea norakhirrudiniana*, *Codonoboea rheophytica* and *Codonoboea sallehuddiniana*, endemism

## Abstract

Of the 92 *Codonoboea* species that occur in Peninsular Malaysia, 20 are recorded from the state of Terengganu, of which 9 are endemic to Terengganu including three new species, *C. norakhirrudiniana* Kiew, *C. rheophytica* Kiew and *C. sallehuddiniana* C.L.Lim, that are here described and illustrated. A key and checklist to all the Terengganu species are provided. The majority of species grow in lowland rain forest, amongst which *C. densifolia* and *C. rheophytica* are rheophytic. Only four grow in montane forest. The flora of Terengganu is still incompletely known, especially in the northern part of the state and in mountainous areas and so, with botanical exploration, more new species can be expected in this speciose genus.

## Introduction

The centre of diversity of the genus *Codonoboea* (Gesneriaceae) is Peninsular Malaysia from where at least 92 species of the 140 named species are known (Lim and Kiew 2014). However, while the west coast of Peninsular Malaysia is relatively well-collected and from where 35 species were described (Kiew and Lim 2011), the east coast is comparatively poorly known with, for example, prior to this study, just six described from Terengganu. The total now stands at 20 species.

**Figure 1.**
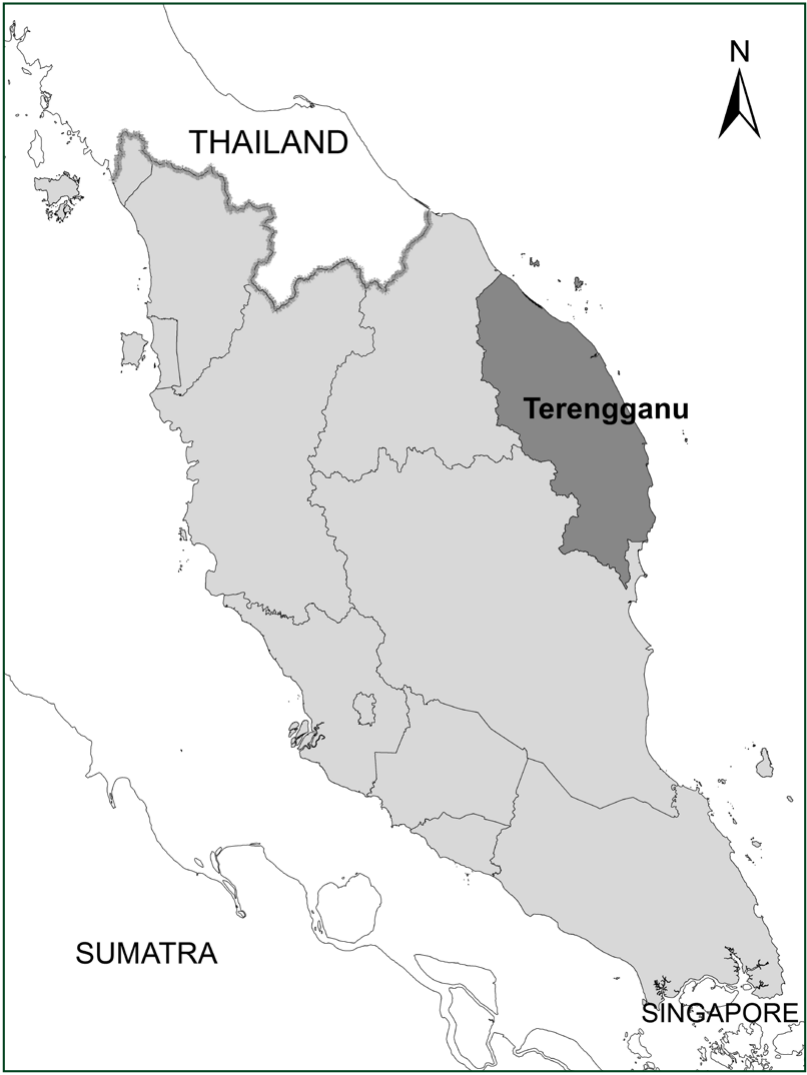
Peninsular Malaysia showing Terengganu State.

**Figure 2.**
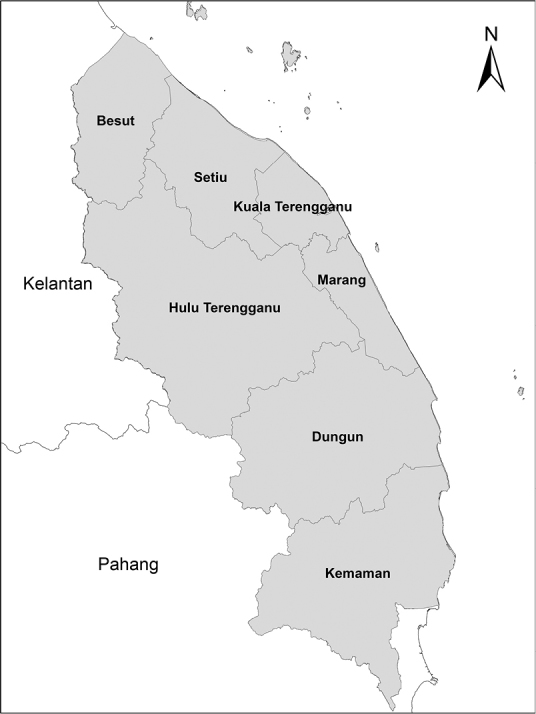
The districts in Terengganu, Peninsular Malaysia.

Terengganu (Figures 1 and 2), one of the eleven states in Peninsular Malaysia, lies on the east coast with a coastline 320 km long, facing the South China Sea. It covers about 150 km^2^ and is bounded on the west by the Terengganu Range, a low range of granite hills, the highest of which is Gunung Lawit at 1,519 m elevation. The hills are covered by dipterocarp forest, below 400 m by lowland dipterocarp forest, to 750 m by hill dipterocarp forest merging into lower montane forest at about 1,200 m, above which the mountain peaks are covered by upper montane forest (Ummul-Nazrah et al. 2011). The hills fall steeply with the smaller tributaries, the saraca-streams (*Saraca cauliflora*) of Corner (1988), at first rocky and torrential but, as the ground levels, they become broader though still rocky and are shaded by neram trees, *Dipterocarpus oblongifolius*, that arch over the river. Rheophytes are characteristic of neram rivers where they gain a foothold in cracks in rocky bedrock or on large boulders or on sandy or pebbly spits that are deposited by periodic floods. Neram rivers cease at the tidal reach where the water becomes brackish. Along the coast and about 25–35 km inland, kapur forests, *Dryobalanops aromatica*, predominate and on sandy, frequently waterlogged and podzolic soil, tropical heath forest develops.

The first *Codonoboea* species described from Terengganu, *C. densifolia* (Ridl.) C.L.Lim (originally described as *Paraboea caerulea* Ridl.) was collected in 1904 by E.Rostados from Bukit Bandi [Bundi], Kemaman District, in south Terengganu where there was a tin mine. In 1932 and 1935, E.J.H. Corner collected from Sungai Nipah, Kemaman District and many of the species he collected later proved to be new to science (Kiew 1991a), such as *C. floribunda* (M.R.Hend.) C.L.Lim (Henderson 1933) and *C. corneri* (Kiew) Kiew (Kiew 1990), indicative of the rich biodiversity of the area. In 1986, R. Kiew with S. Anthonysamy explored northern Terengganu discovering two new species, *C. anthonyi* (Kiew) C.L.Lim from Ulu Besut, Besut District and *C. leiophylla* (Kiew) C.L.Lim from Ulu Sungai Setiu, Setiu District (Kiew 1992) and, later, *C. miniata* (Kiew) C.L.Lim was described from Bukit Bauk, Dungun District (Kiew 1995).

One of the objectives of the Flora of Peninsular Malaysia project (Kiew and Rafidah 2007) is to explore and collect from botanically poorly known regions. For this reason, Terengganu has been a focus and the botany team of the Kepong Herbarium, Forest Research Institute Malaysia, has made regular visits to Terengganu, which has led to the discovery of several new species, including *C. personatiflora* (Kiew & Sam, 2012) and the two new species described below as *C. rheophytica* and *C. sallehuddiniana*. The expedition to Gunung Padang, Hulu Terengganu District, revealed *C. padangensis* (Kiew, 2011) and intensive collecting in the Tembat Forest Reserve, Hulu Terengganu District, prior to its being clear-felled for an extension to the Kenyir Dam, revealed a further two new species, *C. tembatensis* (Kiew, 2014) and the new species, *C. norakhirrudiniana*, described below.

Exploration of the Terengganu flora has led, not only to the discovery of the three new species described below, but also to 12 other *Codonoboea* species described from other states being documented from Terengganu (Table 1), bringing the total presently recorded from Terengganu to 20, of which 9 species (Table 2) are endemic to Terengganu. *Codonoboea anthonyi* and *C. leiophylla*, first described from Terengganu, have since been found in SE Kelantan.

**Table 1.** Distribution of Terengganu *Codonoboea* species that occur in other states (Joh – Johor; Ked – Kedah; Kel – Kelantan; Mel – Melaka; Pah – Pahang; Per – Perak; Sel – Selangor; Ter – Terengganu).

Species	States	Districts in Terengganu
*C. anthonyi*	Kel, Ter	Besut
*C. atrosanguinea*	Kel, Pah, Ter	Dungun, Hulu Terengganu
*C. codonion*	Pah, Ter	Dungun, Kemaman, Hulu Terengganu
*C. densifolia*	Joh, Pah,Ter	Kemaman
*C. grandifolia*	Pah, Ter	Hulu Terengganu (Gunung Padang)
*C. leiophylla*	Kel, Ter	Besut, Setiu, Hulu Terengganu
*C. platypus*	Most states	Dungun, Kemaman, Hulu Terengganu, Setiu
*C. puncticulata*	Joh, Pah, Ter,	Dungun, Kemaman, Hulu Terengganu
*C. quinquevulnera*	Joh, Kel, Mel, Pah, Sel, Ter	Besut, Hulu Terengganu
*C. rugosa*	Ked, Kel, Pah, Per, Ter	Kemaman, Hulu Terengganu
*C. salicinoides*	Joh, Kel, Pah, Ter	Besut, Dungun, Kemaman

**Table 2.** Distribution of *Codonoboea* species endemic in Terengganu.

Species	District	Locality
*C. corneri*	Dungun, Kemaman	Pasir Raja (FRI 65593), Sg. Nipah
*C. floribunda*	Dungun, Kemaman	Jerangau, Sg Nipah
*C. miniata*	Dungun	Bkt. Bauk, Bkt. Chabang
*C. norakhirrudiniana*	Hulu Terengganu	Tembat
*C. padangensis*	Hulu Terengganu	Gunung Padang
*C. personatiflora*	Dungun, Kemaman, Hulu Terengganu, Setiu	Jengai, Sg. Nipah, Ulu Telemong, Ulu Setiu
*C. rheophytica*	Dungun	Rasau Kertih
*C. sallehuddiniana*	Dungun	Sg Loh, Jerangau, Pasir Raja
*C. tembatensis*	Hulu Terengganu	Tembat

Three new species are described here. There are undoubtedly more species that await discovery as many areas remain to be explored. Besides a checklist for Terengganu *Codonoboea*, a key is provided to facilitate their identification.

## Materials and methods

Specimens, including type specimens of Peninsular Malaysian *Codonoboea* species in the herbaria at BM, K, KEP, KLU, SING and UKMB (acronyms follow Thiers 2019), were examined. All specimens cited were seen by the authors. Literature relevant to the region (West Malesia and Thailand) was consulted. Conventional methods employed in herbarium taxonomy were applied in this study. All measurements were taken from dried herbarium specimens. The spelling of localities follows Hamidah et al. (2011).

## Results

### Key to *Codonoboea* species in Terengganu

**Table:** 

1	Leaves very narrow, to 2 cm wide and at least 6.5 times longer than wide	**2**
–	Leaves more than 2 cm wide and less than 5 times longer than wide	**3**
2	Lamina 6.6–8.2 times longer than wide (4–15.6 × 0.6–1.9 cm), margin entire. Inflorescences 1–4-flowered. Capsule 1.6–2.9 cm long	***C. densifolia***
–	Lamina 14–17 times longer than wide (11.5–22 × 0.8–1.3 cm), margin crenate. Flowers single. Capsule 2.8–4.3 cm long.	***C. rheophytica***
3	Flowers single	**4**
–	Inflorescences with 2–18 flowers	**12**
4	Flowers epiphyllous. Leaves spaced on stem	***C. corneri***
–	Flowers in leaf axils. Leaves crowded towards top of stem	**5**
5	Rosette herbs to 2–19 cm tall. Leaf lamina to 16 × 5 cm long, lateral veins 7–15 pairs. Flowers campanulate, to 2 cm long	**6**
–	Robust, unbranched herbs 40–100 cm tall. Leaf lamina more than 15 × 4 cm, lateral veins 20 pairs or more. Flowers trumpet-shaped, 3–4 cm long	**8**
6	Lamina glabrous above, margin more-or-less entire. Capsule conspicuously upturned	***C. leiophylla***
–	Lamina hairy above, margin minutely serrate. Capsule straight	**7**
7	Lamina base rounded or cordate, flat above, often with a broad silver-grey band along the midrib, apex rounded. Calyx 1–1.7 mm long; corolla tube shorter (4–5.5 mm) than the lobes (6–7.3 mm). Capsule 2–3 cm long	***C. puncticulata***
–	Lamina base narrowed, single hairs raised on protruding aeroles, plain-coloured, apex acute. Calyx 3–4.5 mm long; corolla tube longer (12–14 mm) than lobes (4–6.5 mm). Capsule 1.5–2.5 cm long	***C. padangensis***
8	Leaves petiolate, petiole to 3 cm long; lateral veins to 20 pairs. Flowers completely bright vermillion. Capsules to 3–4 cm long	***C. miniata***
–	Lamina sessile; lateral veins 25 pairs or more. Flowers white or purplish or if corolla lobes red, then tube is yellow. Capsules 3.5–12 cm long	**9**
9	Lamina with tertiary venation forming a pattern of rectangles. Capsules shorter and thicker, 3.5–4.5 cm long, 2–3 mm diameter	***C. rugosa***
–	Lamina with tertiary venation forming a pattern of polygons. Capsules 5–12 cm long and slender ca. 1.5 mm diameter	**10**
10	Leaves with upper lamina surface softly and finely velvety; margin minutely serrate, lateral veins ca. 25–27 pairs. Corolla tube pale cream or orange outside, lobes deep crimson, throat golden yellow. Capsules (7.5–)9–12 cm long	***C. atrosanguinea***
–	Leaves with upper surface coarse and hispid or densely silky, margin coarsely serrate; lateral veins 33–50 pairs. Corolla white or white tinged purple, lobes sometimes deep purple. Capsule 5–9 cm long	**11**
11	Corolla white, lobes deep purple. Lamina 14–27 × 4.5–7 cm, base often broadly winged and deeply laciniate, upper surface softly and densely silky. Capsule ca. 5 cm long	***C. quinquevulnera***
–	Corolla white tinged purple, rarely pure white, lobes not a deeper purple than tube. Lamina (15–)26–40(–55) × (5.5–)7–9(–15) cm, base with a narrow wing, upper surface coarsely hispid. Capsule 7–9 cm long	***C. platypus***
12	Corolla 15–40 mm long	**13**
–	Corolla 7–15 mm long	**16**
13	Inflorescences short or subsessile, (1–)2–3 flowered. Corolla completely white except for lemon-yellow nectar guides. Usually branching at the base and forming clumps. Petioles slender 3–5.5 cm long, lamina 9–11 cm long, 2–3 times longer than petiole, softly hairy. Capsules 1.5–1.8 cm long, broad at base, 3.5–5 mm thick	***C. tembatensis***
–	Inflorescences with a long peduncle more than 11 cm long, many-flowered. Flowers purple or rosy purple. Not clump forming. Lamina 4–9 times longer than petiole. Capsules 3–8 cm long, slender, to 1–2 mm thick	**14**
14	Petioles 5–6 cm long, lamina broader, ca. 28 × 9 cm. Flowers 35–40 mm long. Capsules 6–8 cm long	***C. grandifolia***
–	Petioles 1.3–4 cm long, lamina 9–1 × 4–7 cm or 15–34 × 4–8 cm. Flowers 15–20 mm long. Capsules 3–4.5 cm long	**15**
15	Leaf lamina shorter 9–17 × 4–7 cm, drying greenish-brown above, margin deeply serrate. Corolla 15–16 mm long, rosy purple, tube slender, 4–6 mm diameter at mouth, lobes reflexed	***C. sallehuddiniana***
–	Leaf lamina 15–34 × 4–8 cm, drying deep purplish-brown above, margin minutely crenate. Corolla ca. 20 mm long, pale cream with pale pink lobes, tube 6–9 mm diameter at mouth, lobes personate	***C. personatiflora***
16	Lamina narrow, 9–15 × 2.3–3 cm, ca. 4.5 times longer than wide; petiole and midrib sometimes drying wrinkled with transverse ribs. Peduncle and pedicels finely slender and thread-like. Capsule short 1.2–1.4 cm long	***C. salicinoides***
–	Lamina 10–21 × 4–9 cm, ca. 2.5 times longer than wide; petiole and midrib not drying wrinkled with transverse ribs. Capsule 1.5–2.3 cm long	**17**
17	Inflorescences with 8–18 flowers. Corolla 2.5–9 mm long. Stem to 20 cm tall	**18**
–	Inflorescences with (2–)3–4 flowers. Corolla 9–12.5 mm long. Stem robust 40–60 cm tall	**19**
18	Corolla 7–9 mm long, calyx 3–5 mm long. Lamina base narrowed then abruptly rounded	***C. floribunda***
–	Corolla 2.5–4 mm long, calyx 1–1.5 mm long. Lamina base narrowed and cuneate	***C. codonion***
19	Stem and petioles glabrescent. Peduncle 6–8.5(–11) cm long. Corolla pale purple with upper lobes deep purple	***C. anthonyi***
–	Stem and petioles with persistent dense matted pubescence. Peduncle 4.5–6 cm long. Corolla uniformly purple	***C. norakhirrudiniana***

### New species

#### Codonoboea norakhirrudiniana

Taxon classificationPlantaeLamialesGesneriaceae

Kiew sp. nov.

BA5F4105DA975B478F30EC64AE0EE49C

urn:lsid:ipni.org:names:77201449-1

Figure 3

##### Diagnosis.

*Codonoboea norakhirrudiniana* is most similar to *C. anthonyi* (Kiew) C.L.Lim in its 12–19 cm-long oblanceolate leaf lamina that is glabrous above and is narrowed to the base; in its 3–4-flowered inflorescences with peduncles less than 10 cm long, but it is distinct in being a robust herb with a stem to 60 cm tall and 6–7 mm thick (not to 40 cm tall and to 6 mm thick in *C. anthonyi*), the stem and petioles with persistent dense pubescence (not glabrescent), the lower leaves are distant (not with all leaves in a tuft at apex), the peduncle is 4.5–6 cm long (not 6–8.5(–11) cm long) and the flowers are uniformly pale lavender (not pale lavender with deep purple upper lobes).

##### Type.

Peninsular Malaysia. Terengganu, Hulu Terengganu, Tembat Forest Reserve, 5°12.51'N 102°34.22'E, 2 April 2010 Mohd Hairul, M.A. et al. FRI 70907 (holotype KEP; iso. SAN).

##### Description.

Robust, erect, unbranched herb. *Stem* woody, outer layer corky, 16–30(–60) cm tall, 6–7 mm diameter; stem and petiole with persistent, thick matted pubescence. *Leaves* opposite, in a tuft at the top of the stem, lower pairs equal and 0.5–1(–3) cm apart; petioles stout, 0.7–1.3 cm long, deeply grooved above; lamina oblanceolate to narrowly oblanceolate, thinly leathery, glabrous above, 12.5–20 × 3–6 cm, matt, dark green above, whitish-green beneath, drying dark brown above, narrowed into the petiole, margin minutely dentate, teeth to 1.5 mm long, apex acute; midrib and veins in life impressed above, prominent beneath, lateral veins 13–21 pairs; beneath midrib hispid, lateral veins and margin densely and minutely hispid.

*Inflorescence* a pair-flowered cyme with (2–)3–4 flowers, erect from the upper leaf axils, ca. 3 produced from a single leaf axil and the base of the petiole (epiphyllous), peduncle rosy purple or maroon, 4.5–6 cm long, bract pair pale green, linear, 5–8 × ca. 2 mm, pedicels maroon, purple or brown, 5–9 mm long; peduncle, pedicel and calyx densely hispid, hairs non-glandular. *Flowers*: buds white; calyx brownish-green, 5-lobed divided almost to the base, lobes narrow, 4–4.5 × 0.75 mm, apex acute, keeled, pubescent outside with hairs to 1 mm long, glabrous inside; corolla campanulate, plain pale lavender, 9–13 mm long, throat white inside, minutely pubescent outside, glabrous inside, tube 5–7.5 × 3.5–4 mm, lobes 5, bluntly rounded, upper two, 3.5–5 × 4–5 mm, strongly reflexed, lateral lobes ca. 4 × 4–5 mm, median lobe ca. 4.5–5 × 5–6 mm; stamens 2, joined at corolla base and included in corolla tube, filaments white, stout or expanded at the base, 2–2.5 × 0.5–1.5 mm, anthers white, broadly sagittate, 1.5–1.75 × ca. 1.5 mm, cohering face-to-face, staminodes 2, vestigial, ca. 0.75 mm long; nectary annular, minute, ca. 0.2 mm high; ovary and style densely and minutely pubescent, ovary white, 4–4.5 mm long, style white, ca. 4 mm long projecting beyond the upper corolla lobes, stigma rounded, minute, ca. 0.5 × 1mm. *Fruits* slender, cylindric, 24–40 × 1–2 mm, dehiscing along the upper suture; pedicel 7–15 mm.

**Figure 3.**
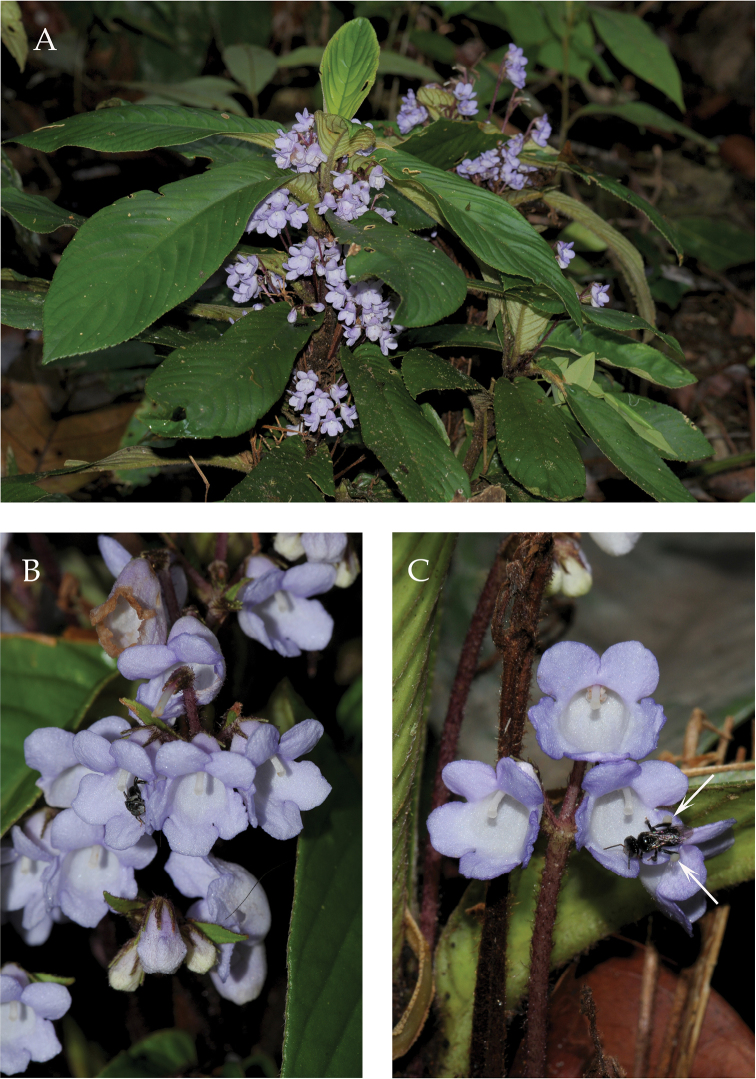
*Codonoboea norakhirrudiniana* Kiew, sp. nov. **A** habit **B** flowers **C** trigona bee leaving flower with full pollen baskets indicated by arrows. (Photographs by Ong Poh Teck).

##### Distribution.

Endemic in Terengganu, Peninsular Malaysia, known only from Tembat Forest Reserve, Hulu Terengganu District.

##### Ecology.

Common in primary or logged-over lowland or hill dipterocarp forest at 390–420 m altitude, on top of ridges or above streams. Flowering gregariously with flowers and fruits on the same plant in March–April and July.

##### Etymology.

Named for Dato’ Indera Hj. Nor Akhirrudin bin Mahmud, formerly Director-General of the Forestry Department Peninsular Malaysia, a strong advocate of conservation, who introduced the system of raising the level of protection by the designation of forest reserves as High Conservation Value Forests.

##### Other specimens examined.

Terengganu: Hulu Terengganu, Tembat Forest Reserve – Mohd Hairul, M.A. et al. FRI 60919 (KEP), Mohd Hairul, M.A. et al. FRI 70907 (KEP, SAN), Kamarul, M. et al. FRI 66338 (KEP), Kamarul, M. et al. FRI 67125 (KEP, L, SAN, SING), Kamarul, M. et al. FRI 67147 (KEP, L, SAN, SING), Nor Ezzawanis, A.T. et al. FRI 58126 (KEP, K, SAN, SAR, SING), Ummul Nazrah, A.R. et al. FRI 57005 (KEP, SING), Ummul Nazrah, A.R. et al. FRI 57035 (KEP).

##### Notes.

This species belongs to the Boeopsis group of *Codonoboea* species that have a short campanulate flower with large anthers prominent in the mouth of the corolla and with a minute nectary, all typical characters of a pollen flower (Lim 2014). Molecular analysis demonstrates that this species (labelled *Codonoboea* sp. nov. 5 in phylogenetic tree) clusters with *C. anthonyi* and *C. leiophylla* but is distinct from them (Lim and Kiew 2014).

It grows in the Tembat Forest Reserve. In 2010, the KEP botany team carried out an intensive collecting programme in the area that was designated for clear-felling for the extension to the Kenyir Hydroelectric Dam in an effort to provide a permanent record of this little known forest. As a result, several new species were discovered, such as two species of *Codonoboea*, *C. norakhirrudiniana* and *C. tembatensis* (Kiew, 2014). Although both these species were quite common and widespread within the Tembat Forest Reserve, they have not been found in other forests in Terengganu. The Tembat Forest Reserve has since been clear-felled for the extension to the Kenyir Hydroelectric Dam. The current status of their populations is not known but, because of their restricted distributions, there is the possibility that the widespread clear-felling will ultimately result in their extinction.

#### Codonoboea rheophytica

Taxon classificationPlantaeLamialesGesneriaceae

Kiew sp. nov.

79A461B0C59A5BD5B2826A868EB3BA4A

urn:lsid:ipni.org:names:77201450-1

Figure 4

##### Diagnosis.

In its extremely narrow leaves, less than 2 cm wide and more than eight times longer than wide, it resembles the other two Peninsular Malaysian rheophytic *Codonoboea* species, *C. densifolia* and *C. salicina*. It shares with *C. salicina* the serrate leaf margin, but it differs in its leaves that have more lateral veins (33–36 pairs vs.13–18 pairs in *C. salicina*) and from *C. densifolia* that has a leaf with an entire margin. *Codonoboea rheophytica* differs from both in its sessile leaves (vs. shortly petiolate, petioles 0.5–1.5 cm long), single flowers (not in inflorescences with 2–4 flowers) and longer corolla tube 30–40 × 7–12 mm (vs. 8–9 × 4.5 mm in *C. densifolia* and 3.3 × 3.7 mm in *C. salicina*) and longer fruit 3–4.5 cm long (vs. 1.5–3 cm long). Amongst species with similar solitary flowers with a large trumpet-shaped corolla, included stamens, annular nectary ca. 1 mm tall and a large peltate stigma and sessile leaves with a serrate margin, it most resembles *Codonoboea crinita* that has relatively narrow leaves (13.5–)18(–24) × (3–)3.5(–4.5) cm. It differs from *C. crinita* in its much narrower leaves (0.8–1.3 cm wide and 14–17 times longer than wide), in its lamina that is glabrous above, except for the midrib (vs. densely hairy in *C. crinita*), 33–34 pairs of lateral veins, (vs. 22–28(–34) pairs) and shorter fruit 2.8–4.3 cm long (vs. 5.5–8 cm long). In addition, the leaves of *C. crinita* are usually deep purple beneath and have a broad silver-grey band along the midrib on the upper surface. *Codonoboea crinita* grows on soil and is very rarely recorded as a lithophyte and, in spite of being a common and widespread species, it has never been recorded as growing on rocks in rivers or even on river banks.

##### Type.

Peninsular Malaysia. Terengganu, Dungun District, Rasau Kerteh Forest Reserve. 4°35.52'N, 103°17.47'E, 20 Oct 2002, Sam, Y.Y., Angan, A., Mustafa, D. FRI 47176 (holotype KEP; iso: SAN).

##### Description.

Rheophyte, *stem* erect, unbranched, woody, 12–23 cm tall, 5–6 mm diameter; apex and developing leaves densely covered in long, glossy hairs. *Leaves* in a dense tuft at the top of the stem, sessile; lamina narrowly lanceolate, glabrescent above, 11.5–22 × 0.8–1.3 cm, in life bullate, margin serrate, teeth ca. 1–1.5 mm long and 1.5–2 mm broad at base; midrib and veins impressed above, forming conspicuous irregular squares or polygons, midrib shortly hispid, beneath midrib and veins prominent and shortly hispid; lateral veins 33–36 parallel pairs, tertiary veins perpendicular and sending fine veins into sinus between two teeth. *Flowers* single from upper surface of the leaf base. Peduncle and pedicel 6–7.5 cm long, sparsely hairy; bract linear, ca. 2 mm long. Indumentum of peduncle, calyx, corolla and ovary of glandular, long- stalked hairs. Calyx 5-lobed, divided to base, 2–3 mm long, base 1 mm wide, densely pubescent; corolla trumpet-shaped, 3.2–4.5 cm long, white, minutely pubescent outside, glabrous inside, tube 3–3.5 cm long, throat yellow inside, mouth ca. 1.2 cm diameter, lobes 5, broadly rounded, upper two 0.7–1.5 × 0.6–1 cm, edge of lobes purple, upper two erect, lower three spreading, 3–3.5 mm long; stamens 2, white, glabrous, included in corolla tube, filaments ca. 10 mm long, anthers white, broadly sagittate, 2.5–3 × ca. 1.3 mm, cohering face-to-face, staminodes 2, finely linear, 2.5–3 mm long; nectary annular, 1–1.3 mm high; ovary and style densely and minutely pubescent, ovary 11–16 × ca. 1.5 mm, style 9–10 mm long, ovary and style densely hairy; stigma large, peltate 1.3–1.7 mm diameter, with large papillose cells. *Fruits* slender, cylindric, 2.8–4.3 cm long, ca. 1.5 mm diameter, minutely hispid, dehiscing along the upper suture.

**Figure 4.**
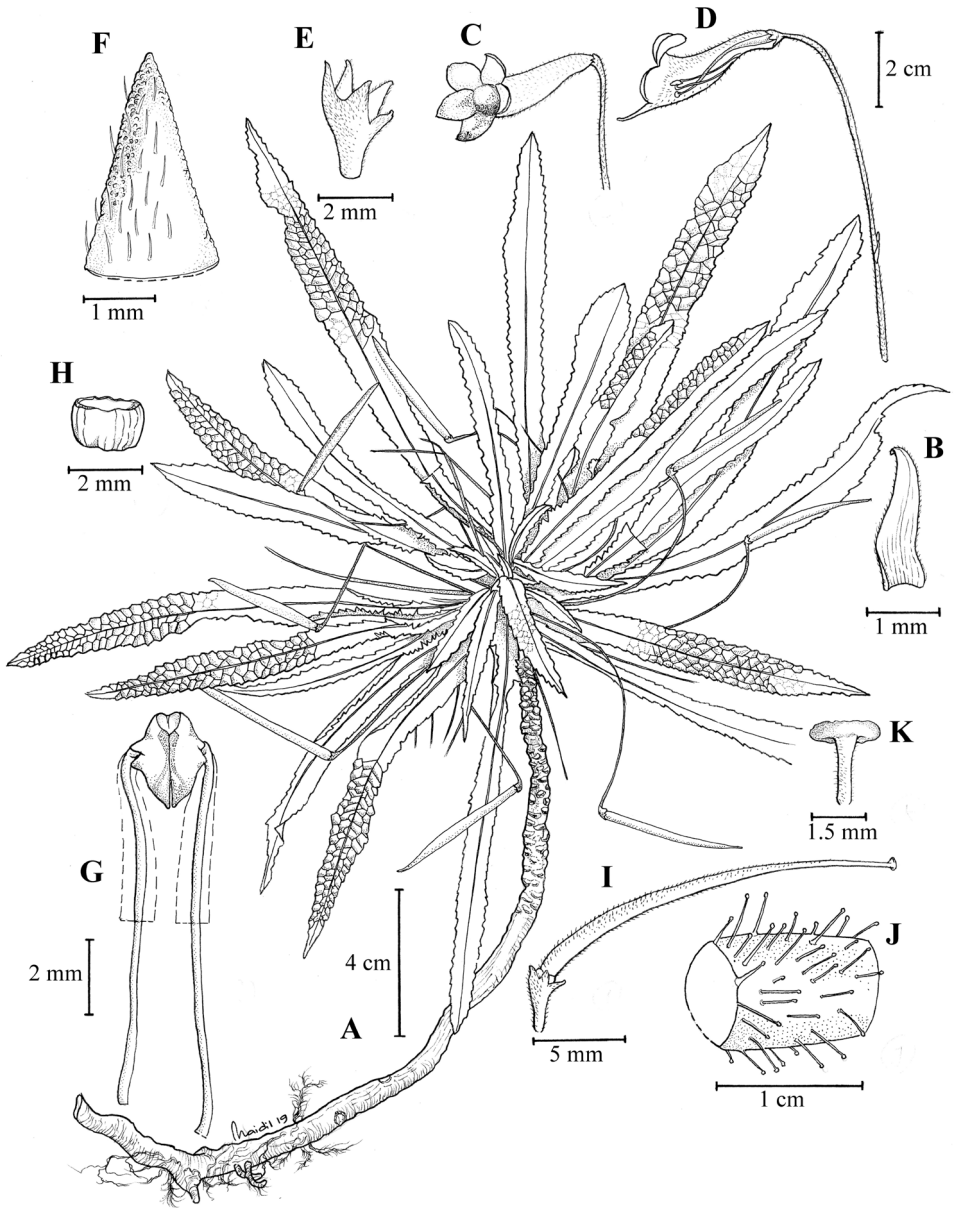
*Codonoboea rheophytica* Kiew, sp. nov. **A** habit **B** bracteole **C** flower **D** flower opened to show position of stamens **E** calyx **F** outer surface of calyx lobe **G** stamens **H** annular nectary **I** young fruit **J** ovary covered in glandular hairs **K** peltate stigma. (All from *FRI 47176*, drawn by Mohamad Aidil Noordin).

##### Distribution.

Endemic in Terengganu, Dungun District, Rasau Kerteh Forest Reserve, known only from the type.

##### Ecology.

Rheophyte clinging onto rock surface along river in regenerated lowland dipterocarp forest at 54 m altitude.

##### Etymology.

Greek – *rheo*, pertaining to flowing water. The rheophytic habit is very unusual in *Codonoboea* species.

##### Notes.

Although *Codonoboea* species are common on rocks close to streams and on river banks, very few, notably *C. densifolia*, *C. salicina* and this new species, grow within the flood zone and are true rheophytes. All three have the typical habit of rheophytes, wiry stem and exceptionally narrow leaves, although *C. densifolia* and *C. salicina* are not obligate rheophytes, since they also grow on steep banks in forest.

Its flower characters place it within the Heteroboea group of species. Other species in this group are large, robust herbs with large, broad leaves quite unlike those of *C. rheophytica*. Molecular analysis confirms that it (labelled as *Codonoboea* sp. nov. 2) falls within this group (Lim and Kiew 2014).

#### Codonoboea sallehuddiniana

Taxon classificationPlantaeLamialesGesneriaceae

C.L.Lim sp. nov.

3D623ED1CC0250348EC60E80679D21BF

urn:lsid:ipni.org:names:77201451-1

Figures 5 , 6

##### Diagnosis.

In its habit (stem unbranched, woody, 10–25 cm long, 3–6 mm thick) and its petiolate, narrowly lanceolate or slightly oblanceolate lamina 9–20 cm long with many, deeply impressed veins and a serrate margin with teeth 3–5 × 1.2–2 mm, *Codonoboea sallehuddiniana* resembles *C. breviflora* (Ridl.) Kiew but it is different in its dichasium with two short branches and flowers in pairs (*C. breviflora* has a one-flowered inflorescence), its large foliose bracts 11–12 mm long (not linear and 5–10 mm long), its rosy purple, narrow corolla 15–16 mm long (not shorter, campanulate, pale purple to white corolla and 10–14 mm long) and shorter fruits 3–3.5 cm long (not 3–5.5 cm long).

##### Type.

Peninsular Malaysia. Terengganu, Dungun, Pasir Raja Forest Reserve, Compartment 5, 4°41.62'N, 102°58.35'E, 28 June 2011 Yao, T.L. & Azril, A. FRI 65593 (holotype KEP, barcode KEP210589; iso: E, SING).

##### Description.

Erect, unbranched herb. *Stem* woody, 15–20(–60) cm tall, 3–6 mm diameter. Indumentum of long ferruginous hairs, on stem and petioles dense and glossy, hairs to 1.5 mm long, grooved midrib on upper lamina surface densely hairy, beneath midrib and veins hairy, denser with hairs to 1.5 mm long on the midrib. *Leaves* at the top of the stem, opposite, pairs equal, to 1.5 cm apart; petioles 1.5–3.5 cm long; lamina lanceolate, glabrous above, 9–17.5 × 4–7 cm, in life bullate, glossy green above, yellowish-green near petiole, pale green beneath, drying reddish-brown, margin glabrous, serrate, teeth falcate, 3–5 × 1.5–2 mm long, apex acute; midrib and veins in life impressed above, prominent beneath; lateral veins 15–24 pairs, glabrous above, a fine vein reaching between every third sinus between the teeth. *Inflorescence* erect, from the upper leaf axils, a pair-flowered dichasium with 2 short equal branches, each with 5–10 pairs of flowers; peduncle slender, maroon or dark purple, 11–16.3 cm long, hairy, hairs to ca. 2 mm long, branches 2–5.5 cm long; bract pair at first purple, then semi-transparent pale green, persistent, sparsely hairy outside, glabrous inside, foliose, 3-veined, lanceolate, 11–12 × 3–4 mm decreasing in size towards the apex, margin distantly serrate; pedicels dark purple, ca. 2 mm long, hairy. *Flowers* held more-or-less horizontally or slightly pendent; calyx dark purple, ca. 3 mm long, densely hairy outside, hairs glandular to 1 mm long, glabrous inside, 5-lobed divided almost to the base; corolla narrowly tubular, 15–17 mm long, 1.5–2 mm diameter at base, slightly dilating and 4–6 mm diameter at the mouth, tube deep rosy purple, whitish towards the mouth with fine deep purple veins that show through in the white throat, minutely hairy outside and on the lower part of floor of throat with two raised yellowish nectar guides running into the mouth and densely covered in glistening hairs, lobes 5, whitish-cream or sometimes green with purple stripe, upper two lobes rounded, 1.5–2.5 × 1–3 mm, reflexed; lower three ca. 2.5–4 × 2–3.5 mm long, spreading, slightly reflexed at tip; stamens 2, filaments slender, ca. 4–5 mm long, glabrous, anthers white, broadly sagittate, 1.5 mm, cohering face-to-face, positioned just inside the corolla tube; nectary annular, ca. 0.7 mm high; ovary and style with densely hairy, ovary violet, ca. 4 mm long, style sparsely minutely pubescent, ca. 3.5 mm long and stigma capitate, white, ca. 0.1 mm long. *Fruits* extremely slender, cylindric, 3–3.5 cm long, ca. 0.7–1 mm diameter, glabrescent, dehiscing along the upper suture; pedicel to 7 mm long.

**Figure 5.**
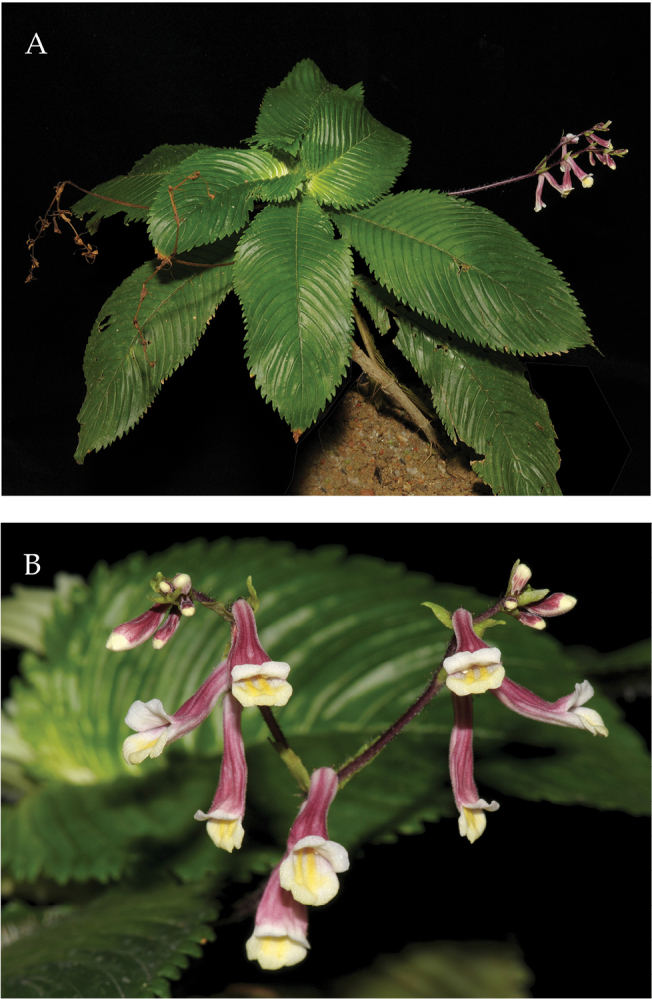
*Codonoboea sallehuddiniana* C.L.Lim, sp. nov. **A** habit **B** Inflorescence. (Photographs by Ong Poh Teck).

**Figure 6.**
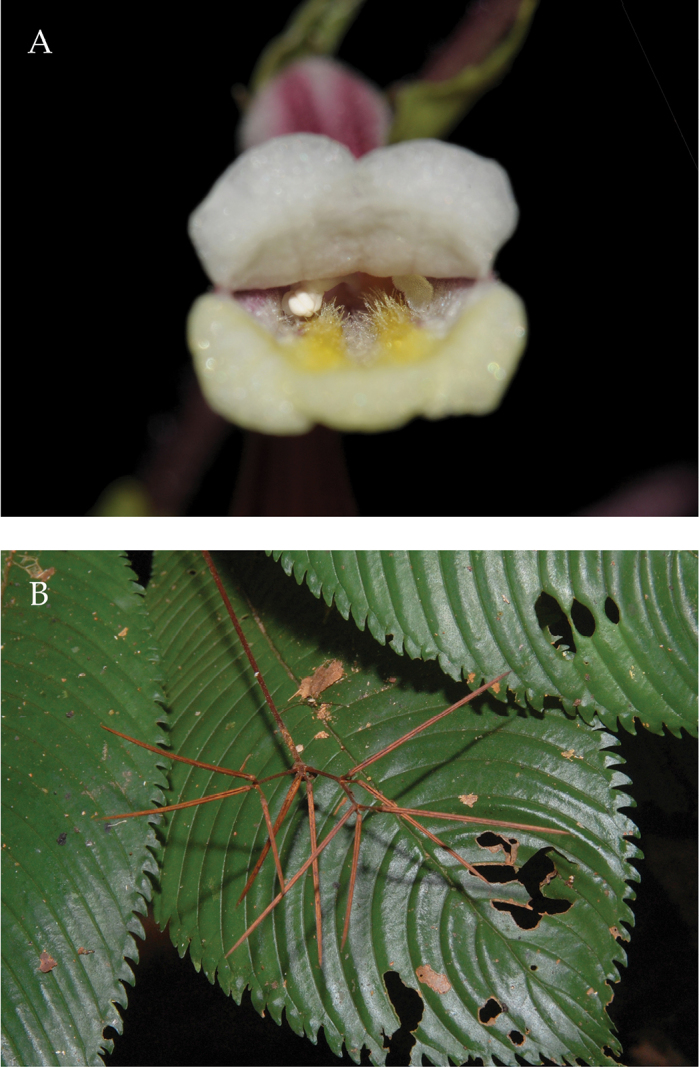
*Codonoboea sallehuddiniana* C.L.Lim, sp. nov. **A** front view of flower **B** Infructescences. (Photographs A by Ong Poh Teck; B by C.L. Lim).

##### Distribution.

Endemic in Dungun District (Jerangau FR, Pasir Raja FR and Sungai Loh), Terengganu, Peninsular Malaysia.

##### Ecology.

Lowland dipterocarp forest, on shaded slopes or top of banks beside old logging road, at 15–50 m altitude.

##### Etymology.

Named for Dato’ Sri Dr Sallehuddin bin Ishak, formerly Federal Lands Commissioner of Malaysia, for his strong support of conservation of karst limestone hills in Perak.

##### Other specimens examined.

Terengganu, Dungun, Jerangau Forest Reserve, Kamarul, M. et al. FRI 67177 (KEP); Ong, P.T. & Rafidah, A.R. FRI 71249 (KEP); Dungun, Sungai Loh, Sam, Y.Y. & Markandan, M. FRI 44400 (KEP), from Sungai Loh cultivated in Forest Research Institute Malaysia, Sam, Y.Y. FRI 47049 (KEP).

##### Notes.

The inflorescence of *Codonoboea sallehuddiniana* is unusual for the genus. The basic inflorescence in *Codonoboea* is a pair-flowered cyme that may be reduced to a single flower (as in *C. breviflora*) or be branched once (a dichasial pair-flowered cyme) or many times to form a thyrse. In *C. sallehuddiniana*, the dichasium has two short branches and, on these, are pairs of short-stalked flowers each subtended by a large foliose bract that decreases in size towards the apex. The flowers are all positioned in the same direction. The particularly long, narrow corolla tube that scarcely dilates and has prominent hairs in the mouth is unusual in *Codonoboea*.

## Discussion

### Habitat

In Terengganu, most *Codonoboea* species have been collected from lowland dipterocarp forest below 250 m elevation in the foothills of the Terengganu Range. Few are found at higher elevations, namely *C. codonion* (to 550 m), *C. puncticulata* (924 m) and, from Gunung (Mount) Padang (at 1,040 m), *C. padangensis* and *C. grandifolia.* However, this is likely an artefact of collecting because very few mountain peaks in Terengganu have been explored botanically. Many species are associated with steep earth slopes, either in forest or on river banks or on rocks but two, *C. densifolia* and *C. rheophytica*, with characteristic narrow leaves, are rheophytes that live on rocks in the flood zone in streams.

No species is recorded from the Marang or Kuala Nerus Districts. This is probably because they are coastal districts and, as yet, no species has been collected from *kapur* forest nor from tropical heath forest, nor from streams and rivers with tidal influence.

*Codonoboea* species are all obligate shade plants that grow in conditions of high humidity. In Terengganu, they are particularly vulnerable because most grow in forest below 250 m elevation and it is this land that is most vulnerable to land use changes. Populations are eliminated by clear-felling forests for palm oil plantations and infrastructure development such as road building or constructing hydroelectric dams. No *Codonoboea* species is weedy and able to adapt to conditions when the tree canopy is opened up from logging or clear-felling (Kiew 2009).

### Distribution

Comparison of species per district illustrates that the northern districts of Besut and Setiu, each with six *Codonoboea* species, are poorer in species compared with 11–12 species recorded for the Dungun, Kemaman and Hulu Terengganu districts. This, however, is likely due to under-collecting; the northern districts are still poorly known. Within the limitation that many areas have still to be explored, particularly in the north and at high elevations, several patterns of distribution can be identified amongst the more widespread species.

Apart from the widespread species (*C. quinquevulnera*, *C. platypus* and *C. rugosa*), the Terengganu flora does not share any species with the Main Range or the west coast states (Table 1). *Codonoboea rugosa* is confined to the northern states and its distribution extends into Thailand. On the east coast, it appears to replace *C. crinita* (Jack) C.L.Lim that is common on the west coast, particularly in the hills. Excluding these widespread species, four species (*C. atrosanguinea*, *C. anthonyi*, *C. leiophylla* and *C. salicinoides*) are shared with Kelantan, four with Pahang (*C. atrosanguinea*, *C. codonion*, *C. grandifolia* and *C. salicinoides*) and two with Johor (*C. densifolia* and *C. puncticulata*). The last two species are much more common in Johor and reach their northern limit in southern Terengganu.

### Endemism

Nationally, the level of endemism in *Codonoboea* (as *Didymocarpus*) is high, 94% (Kiew 1991b) and many species are site endemics. Of the 20 species in Terengganu, only three have distributions extending beyond Peninsular Malaysia. *Codonoboea puncticulata* was recorded from Singapore but that population is now extinct; *C. platypus* is known from Sumatra and Borneo; and *C. platypus* and *C. rugosa* from Peninsular Thailand. Of the nine species endemic to Terengganu (Table 2), *C. padangensi*s and *C. rheophytica* are each at present known from a single population and *C. miniata*, *C. norakhirrudiniana* and *C. tembatensis* from restricted areas.

## Checklist of *Codonoboea* species in Terengganu, Peninsular Malaysia

(Bkt. Bukit (hill); FR Forest Reserve, G. Gunung (mountain), P. Pulau (island), Sg. Sungai (river))

**1. *Codonoboea anthonyi* (Kiew) C.L.Lim**

In Kiew & Lim, Gard. Bull. Sing. 62 (2011) 256. *Basionym: Didymocarpus anthonyi* Kiew, Gard. Bull. Sing. 44 (1992) 24. *Homotypic synonym: Henckelia anthonyi* (Kiew) A.Weber, Beitr. Biol. Pflanzen 70 (1998) 339. *Type*: Peninsular Malaysia, Terengganu, Ulu Besut, *Kiew RK 2700*, 7 May 1988 (holotype KEP; isotypes K, SING).

**Distribution.** Endemic in Peninsular Malaysia – Kelantan (Relai FR) and Terengganu.

**Ecology.** Primary lowland forest to 200 m altitude. Locally common on vertical earth banks or hill slopes by river.

**Terengganu specimens examined.** BESUT DISTRICT: Pelagat FR – *Kiew*, *R. RK 2700* (KEP), *Lim*, *C.L. FRI 64994* (KEP, SAN, SING, L, K, A), *Sam*, *Y.Y. FRI 46648* (KEP), *Sam*, *Y.Y. FRI 47022* (KEP); Ulu Besut FR – *Anthonysamy*, *S. SA 675* (KEP, SING).

**2. *Codonoboea atrosanguinea* (Ridl.) C.L.Lim**

In Kiew & Lim, Gard. Bull. Sing. 62 (2011) 257. *Basionym: Didymocarpus atrosanguineus* Ridl., Trans. Linn. Soc. Ser. 2, Bot.3 (1893) 328, J. Straits Branch Roy. Asiat. Soc. 44 (1905) 47, J. Asiat. Soc. Bengal, Pt. 2, Nat. Hist. 74 (1908) 758, Fl. Malay. Pen. 2 (1923) 518; Kiew, Gard. Bull. Singapore 42 (1989) 49. *Homotypic synonym: Henckelia atrosanguinea* (C.B.Clarke) A.Weber, Beitr. Biol. Pflanzen 70 (1998) 340. *Type*: Peninsular Malaysia, Pahang, Sungai Tahan, *Ridley s.n*. (lectotype SING).

**Distribution.** Endemic in Peninsular Malaysia – Kelantan (Kuala Aring), Pahang (Tahan and Kenyam Valleys) and Terengganu.

**Ecology.** In deep shade in primary lowland forest to 200 m altitude, locally common.

**Terengganu specimens examined.** DUNGUN DISTRICT: Jerangau FR – *Lim*, *C.L. FRI 73027* (KEP); HULU TERENGGANU DISTRICT: Batu Biwa – *Kiew*, *R. RK 2320* (KEP); Sekayu FR – *Anthonysamy*, *S. SA 643* (KEP), *Kiew*, *R. RK 2693* (KEP), *RK 3787* (KEP), *Yao*, *T.L. et al. FRI 77368* (KEP); Sg. Cicir FR – *Julius*, *A. et al. FRI 56113* (KEP); Sg. Perepak FR – *Jutta*, *M. FRI 59559* (KEP, SING, K), *Lim*, *C.L. et al. FRI 52898* (KEP. SAN); Sg. Petuang – *Nor-Ezzawanis*, *A.T. FRI 52288* (KEP), *Phoon*, *S.N. et al. FRI 51979* (KEP, SAN); Tembat FR – *Mohd Hairul*, *M.A. et al. FRI 60958* (KEP), *FRI 72228* (KEP), *Siti-Munirah*, *M.Y. et al. FRI 67864* (KEP, *FRI 67890* (KEP, K); Ulu Telemong FR – *Phoon*, *S.N. et al. FRI 51587* (KEP, KLU), *FRI 51590* (KEP).

**3. *Codonoboea codonion* (Kiew) C.L.Lim**

In Kiew & Lim, Gard. Bull. Sing. 62 (2011) 257. *Basionym: Didymocarpus codonion* Kiew, Gard. Bull. Sing. 42 (1989) 49. *Homotypic synonym: Henckelia codonion* (Kiew) A.Weber, Beitr. Biol. Pflanzen 70 (1998) 342. *Type*: Peninsular Malaysia, Pahang, Kuala Kenyam, *Kiew B.H. RK 1204*, 30 September 1982 (holotype KEP; isotype SING).

**Distribution.** Endemic in Peninsular Malaysia – Pahang (Taman Negara, Gunung Aiis FR) and Terengganu.

**Ecology.** Lowland to hill forest, 15–550 m altitude, on forest floor, slopes, on ridge tops and stream banks.

**Terengganu specimens examined.** DUNGUN DISTRICT: Chemerong FR – *Lim*, *C.L. FRI 64963* (KEP); Jengai FR – *Lim*, *C.L. et al. FRI 72806* (KEP), *Sam*, *Y.Y. & Mustafa*, *D. FRI 47151* (KEP); *Anonymous s.n. (0105938)* (SING); Pasir Raja FR – *Sam*, *Y.Y. & Angan*, *A. FRI 47164* (KEP). KEMAMAN DISTRICT – Bukit Bandi FR – *Sam*, *Y.Y. FRI 47181* (KEP); Sg. Nipah *Chan*, *Y.C. et al. FRI 70608* (KEP, L, SAN, SAR), *Kiew*, *R. RK 2653* (KEP). HULU TERENGGANU DISTRICT – Batu Biwa: *Kiew*, *R. RK 2301* (SING); G. Padang: *Moysey*, *L. 33387* (SING); *FRI 64497* (KEP); Tasik Kenyir: *Lim*, *C.L. FRI 52983* (KEP), *Julius*, *A. FRI 56111* (KEP, SAN, SING, L); Ulu Telemong FR: *Kiew*, *R. RK 5344* (KEP), *Chew*, *M.Y. FRI 51821* (A, KEP, SING).

**4. *Codonoboea corneri* (Kiew) Kiew**

In Kiew & Lim, Gard. Bull. Sing. 62 (2011) 258. *Basionym: Didymocarpus corneri* Kiew, Blumea 35 (1990) 172, figs. 2 & 4. *Homotypic synonym: Henckelia corneri* (Kiew) A.Weber, Beitr. Biol. Pflanzen 70 (1998) 342. *Type*: Peninsular Malaysia, Terengganu, Sg. Nipah *Kiew RK 2655* (holotype L; isotypes K, KEP, SING).

**Distribution.** Endemic in Peninsular Malaysia – Terengganu (Dungun and Kemaman Districts).

**Ecology.** Common on steep earth banks and by streams in lowland forest at ca. 100 m elevation.

**Terengganu specimens examined.** DUNGUN DISTRICT – Pasir Raja FR *FRI 65593*. KEMAMAN DISTRICT – Sg. Nipah, *Corner s.n.* (SING).

**5. *Codonoboea densifolia* (Ridl.) C.L.Lim**

In Kiew & Lim, Gard. Bull. Sing. 62 (2011) 259. *Basionym: Didymocarpus densifolius* Ridl., J. Straits Branch Roy. Asiat. Soc. 44 (1905) 51, J. Asiat. Soc. Bengal, Pt. 2, Nat. Hist. 74 (1908) 761, Fl. Malay Pen. 2 (1923) 521. *Homotypic synonyms: Paraboea densifolia* (Ridl.) M.R.Hend., Gard. Bull. Straits Settlem. 5 (1930) 79. *Henckelia densifolia* (Ridl.) A.Weber, Beitr. Biol. Pflanzen 70 (1998) 343. *Type*: Peninsular Malaysia, Johor, G. Janing [Janeng], *Lake & Kelsall s.n.*, 20 October 1892 (holotype SING). *Heterotypic synonyms: Paraboea caerulea* Ridl., J. Straits Branch Roy. Asiat. Soc. 44 (1905) 66, J. Asiat. Soc. Bengal, Pt. 2, Nat. Hist. 74 (1908) 772, Fl. Malay Pen. 2 (1923) 529; *non Didymocarpus caeruleus* (R.Br.) Koord. – *Didymocarpus azureus* B.L.Burtt, Notes Roy. Bot. Gard. Edinburgh 31 (1971) 44. *Type*: Peninsular Malaysia, Terengganu, ‘Bundi’ [Bukit Bandi], *Rostados* s.n., February 1904 (holotype SING).

**Distribution.** Endemic in Peninsular Malaysia – Johor (Panti FR and Endau-Rompin State Park), Pahang (Pulau Tioman) and Terengganu (Bukit Bandi).

**Ecology.** Lowland to hill dipterocarp forest 60–750 m altitude, locally common on vertical earth bank and rocky stream bank.

**Terengganu specimens examined.** KEMAMAN DISTRICT – Bkt. Bandi [Bundi] *Rostados* s.n. (SING).

**Note.** In Terengganu known only from one old 1904 collection, *Rostados* s.n., that Ridley (1905) described as *Paraboea caerulea.* It is a very distinct narrow-leaved species not likely to be confused with any other species. It is not known if the ongoing tin-mining in 1904 destroyed its habitat, which would explain why it has not been collected since. Rheophytes are particularly vulnerable to habitat disturbance that would cause increased silt disposition, destruction of river banks or the opening of the canopy. It is likely, therefore, that the Bkt. Bandi population has been eliminated. However, it is common in Johor (Kiew, 1987).

**6. *Codonoboea floribunda* (M.R.Hend.) C.L.Lim**

In Kiew & Lim, Gard. Bull. Sing. 62 (2011) 261. *Basionym: Paraboea floribunda* M.R.Hend., Gard. Bull. Sing. 7 (1933) 117. *Homotypic synonyms: Didymocarpus floribundus* (M.R.Hend.) B.L.Burtt, Notes Roy. Bot. Gard. Edinburgh 31 (1971) 44. *Henckelia floribunda* (M.R.Hend.) A.Weber, Beitr. Biol. Pflanzen 70 (1998) 345. *Type*: Peninsular Malaysia, Terengganu, Kemaman, Bkt. Kajang, Sg. Nipah, *Corner 26022*, June 1932 (holotype K).

**Distribution.** Endemic in Peninsular Malaysia, Terengganu (Dungun and Kemaman District).

**Ecology.** Lowland to upper hill dipterocarp forest, 81–152 m altitude, on forest floor and stream banks.

**Terengganu specimens examined.** DUNGUN DISTRICT: Jerangau FR: *Lim*, *C.L. & Nazri*, *A. FRI 65040* (KEP, SAN, SING), *Julius*, *A FRI 56176* (KEP, E, SING). KEMAMAN DISTRICT: Bkt. Kajang – *Kiew*, *R. RK 2686* (SING), *RK 2687* (SING), *RK 2676* (SING), *Corner*, *E.J.H. SFN 30714* (SING), *Imin*, *K. et al. FRI 76175* (KEP); Jeram Tanduk *Lim*, *C.L. et al. FRI 64971* (KEP); Sg. Nipah – *Kiew*, *R. RK 2653* (KEP), *Sam*, *Y.Y. FRI 47190* (KEP), *FRI 47199* (KEP), *FRI 47223* (KEP), *Kiew*, *R. RK 5327* (KEP), *Lim*, *C.L. FRI 64971* (KEP, SAN, SING, L, K, E), *FRI 65153* (KEP); Ulu Bendong – *Corner*, *E.J.H. SFN 30110* (L, SING). Sri Bangun: *Sinclair*, *J. SFN 39863* (SING).

**7. *Codonoboea grandifolia* (Ridl.) Kiew**

In Kiew & Lim, Gard. Bull. Sing. 62 (2011) 261. *Basionym: Paraboea grandifolia* (Ridl.) Ridl., Fl. Malay. Pen. 2 (1923) 531. *Homotypic synonyms: Didymocarpus grandifolius* Ridl., J. Linn. Soc. Bot. 38 (1908) 318 *non Didymocarpus grandifolius* (A.Dietr.) F.G.Dietr. (1834); *Didymocarpus tahanicus* B.L.Burtt, Notes Roy. Bot. Gard. Edinburgh 31 (1971) 46; Kiew, Gard. Bull. Singapore 42 (1989) 61, Malay. Nat. J. 48 (1995) 205; *Henckelia tahanica* (B.L.Burtt) A.Weber, Beitr. Biol. Pflanzen 70 (1998) 357. *Type*: Peninsular Malaysia, Pahang, G. Tahan *Wray & Robinson 5369* (holotype BM; isotype SING).

**Distribution.** Endemic in Peninsular Malaysia, Pahang (G. Tahan) and Terengganu (G. Padang).

**Ecology.** Lower montane forest at 1100–1220 m elevation, in shade on very steep earth slopes.

**Terengganu specimens examined.** Hulu Terengganu DISTRICT: G. Padang – *Moysey*, *L. & Kiah SFN 33924* (SING), *FRI 12700* (KEP).

**Note.** It is a striking species with particularly large leaves and flowers. It only grows on steep earth slopes and appears to be rare and local.

**8. *Codonoboea leiophylla* (Kiew) C.L.Lim**

In Kiew & Lim, Gard. Bull. Sing. 62 (2011) 264. *Basionym: Didymocarpus leiophyllus* Kiew, Gard. Bull. Sing. 44 (1992) 28. *Homotypic synonym: Henckelia leiophylla* (Kiew) A.Weber, Beitr. Biol. Pflanzen 70 (1998) 348. *Type*: Peninsular Malaysia, Terengganu, Ulu Setiu *Kiew RK 2265*, 28 April 1986 (holotype KEP; isotype SING).

**Distribution.** Endemic in Peninsular Malaysia, Kelantan (Kuala Aring FR) and Terengganu (Besut, Hulu Terengganu and Setiu districts).

**Ecology.** Lowland, locally common on vertical earth banks, hill slopes by river, to 100 m altitude.

**Terengganu specimens examined.** BESUT DISTRICT: G. Tebu FR – *Lim*, *C.L. FRI 64998* (KEP, SAN, SING). HULU TERENGGANU DISTRICT: G. Lawit – *Kiew*, *R. RK 2272* (SING). SETIU DISTRICT: Ulu Setiu FR – *Anthonysamy*, *S. SA 670* (KEP), *SA 662* (KEP, SING), *Kiew*, *R. RK 2265* (KEP, SING), *Sam*, *Y.Y. FRI 44386* (KEP, KEP, L, SAN, SAR), *Sam*, *Y.Y. FRI 46650* (KEP, SAN), *Mohd Shah MS 3509* (SING), *Anthonysamy*, *S. SA 718* (SING), *Lim*, *C.L. FRI 64951* (KEP), *FRI 64991* (KEP).

**9. *Codonoboea miniata* (Kiew) C.L.Lim**

In Kiew & Lim, Gard. Bull. Sing. 62 (2011) 266. *Basionym: Didymocarpus miniatus* Kiew, Novon 5 (1995) 40. *Homotypic synonym: Henckelia miniata* (Kiew) A.Weber, Beitr. Biol. Pflanzen 70 (1998) 350. *Type*: Peninsular Malaysia, Terengganu, Bkt. Bauk (holotype KEP; isotypes L, SING).

**Distribution.** Endemic in Peninsular Malaysia, Terengganu, Dungun District (Bkt. Bauk area).

**Ecology.** In coastal forest on the foothills of low sandstone hills, on slopes above small swampy areas or by seasonal streams.

**Terengganu specimens examined.** DUNGUN DISTRICT: Bkt. Bauk – *Anthonysamy*, *S. SA 602* (KEP), *Kochummen*, *K.M. KEP 9491* (KEP); Bkt. Chabang – *Davison*, *G. GD5* (KEP).

**10. *Codonoboea norakhirrudiniana* Kiew (see above)**

**11. *Codonoboea padangensis* Kiew**

Malay. Nat. J. 63 (2011) 661. *Type*: Peninsular Malaysia, Terengganu, Hulu Terengganu District, G. Padang *Ong et al. FRI 66754* (holotype KEP; isotypes E, K, L, SAR, SING).

**Distribution.** Endemic in Peninsular Malaysia, Terengganu (G. Padang).

**Ecology.** Locally common on steep slopes in lower montane forest at about 1040 m elevation.

**Terengganu specimens examined.** Known only from the type population.

**12. *Codonoboea personatiflora* Kiew & Y.Y.Sam**

Phytokeys 18 (2012) 62. *Type*: Peninsular Malaysia, Terengganu, Kemaman District, Sg. Nipah, Bukit Kajang, *Corner SFN 30540* (holotype SING; isotypes K, L, E, SAR).

**Distribution.** Endemic in Peninsular Malaysia, Terengganu (Dungun, Kemaman, Hulu Terengganu and Setiu districts).

**Ecology.** In primary or logged-over lowland mixed dipterocarp forest at low altitudes (below 100 m), on shaded hillsides or slopes, often above streams.

**Terengganu specimens examined.** Dungun district: Jengai FR – *Anon. s.n.* 15 Mar 1998 (SING), Compartment 52 *Sam*, *Y.Y. FRI 47153* (KEP, SAN). Hulu Terengganu district: Ulu Telemong FR – *Kiew*, *R. RK 5339* (KEP, K, SAR); Ladang Ternakan, Tersat – 31 Oct 2009, *Kamarul*, *M. FRI 67168* (KEP, SING). Kemaman district: Sg. Nipah FR, Jeram Tanduk – *Sam*, *Y.Y. FRI 47197* (KEP, SING). SETIU DISTRICT: Ulu Setiu FR – *Sam*, *Y.Y. FRI 44395* (KEP).

**13. *Codonoboea platypus* (C.B.Clarke) C.L.Lim**

In Kiew & Lim, Gard. Bull. Sing. 62 (2011) 267. *Basionym: Didymocarpus platypus* C.B.Clarke, in A.DC. & C.DC., Mongor. Phan. 5, 1 (1883) 94; Ridley, J. Straits Branch Roy. Asiat. Soc. 44 (1905) 46, J. Asiat. Soc. Bengal, Pt. 2, Nat. Hist. 74 (1908) 757, Fl. Malay. Pen. 2 (1923) 517. *Homotypic synonym: Henckelia platypus* (C.B.Clarke) A.Weber, Beitr. Biol. Pflanzen 70 (1998) 352. *Type*: Peninsular Malaysia, Melaka, *Griffith 3825* (lectotype K).

**Distribution.** Sumatra, Peninsular Thailand and throughout Peninsular Malaysia.

**Ecology.** The commonest *Codonoboea* in Peninsular Malaysia in shaded primary lowland and hill dipterocarp forest to 1000 m altitude. Locally common on vertical earth banks or hill slopes by river.

**Terengganu specimens examined.** DUNGUN DISTRICT: Jengai FR – *Julius*, *A. et al. FRI 57761* (KEP), *Lim*, *C.L. et al. FRI 72830* (KEP); Jerangau FR – *Lim*, *C.L. et al. FRI 65114* (KEP), *Mohd Hairul*, *M.A. et al. FRI 69951* (KEP). *FRI 69988* (KEP). KEMAMAN DISTRICT: Bukit Kajang – *Imin*, *K. et al. FRI 76168* (KEP); Jeram Tanduk – *Lim*, *C.L. et al. FRI 64977* (KEP), *Sam*, *Y.Y. FRI 47196* (KEP); Sg. Nipa – *Kiew*, *R. RK 2666* (KEP). SETIU DISTRICT: Ulu Setiu FR – *Sam*, *Y.Y. FRI 44394* (KEP). Hulu Terengganu District: Tembat FR – *Mohd. Hairul*, *M.A. FRI 60929* (KEP), *Sam*, *Y.Y. FRI 50341* (KEP), *FRI 50367* (KEP), *Siti-Munirah et al. 67875* (KEP); G. Padang – *Ong*, *P.T. et al. FRI 67761* (KEP).

**14. *Codonoboea puncticulata* (Ridl.) C.L.Lim**

In Kiew & Lim, Gard. Bull. Sing. 62 (2011) 268. *Basionym: Didymocarpus puncticulatus* Ridl., J. Linn. Soc. 32 (1896) 510, J. Straits Branch Roy. Asiat. Soc. 44 (1905) 55, J. Asiat. Soc. Bengal, Pt. 2, Nat. Hist. 74 (1908) 763, Fl. Malay Pen. 2 (1923) 522; Kiew, Malay Nat. J. 41 (1987) 220. *Homotypic synonym: Henckelia puncticulata* (Ridl.) A.Weber, Beitr. Biol. Pflanzen 70 (1998) 353. *Type*: Peninsular Malaysia, Johor, G. Panti, *Ridley s.n.*, December 1892 (holotype SING; photo K). *Heterotypic synonym: Didymocarpus perditus* Ridl., J. Straits Branch Roy. Asiat. Soc. 44 (1905) 54, J. Asiat. Soc. Bengal, Pt. 2, Nat. Hist. 74 (1908) 763, Fl. Malay Pen. 2 (1923) 522. *Type*: Singapore, Selitar, *Ridley s.n.*, 3 Nov 1889 (holotype SING).

**Distribution.** Singapore (extinct) and Peninsular Malaysia (Johor, Pahang and Terengganu).

**Ecology.** Lowland to hill forest, 20–924 m altitude, on various habitats, from shaded earth bank, river banks, rock surface in forest floor, river or by waterfall.

**Terengganu specimens examined** Dungun district: Bkt. Bauk FR – *Anthonysamy*, *S. SA 601* (KEP), *Weber*, *A. UPM 3420* (KEP), *Davison*, *G.W.H. GD 6* (KEP, SING), *Kochummen*, *K.M. FRI 2592* (KEP), *Sam*, *Y.Y. FRI 44397* (KEP, SAN, SAR), *FRI 47170* (KEP), *Saw*, *L.G. FRI 44888* (KEP, SING), *Sam*, *Y.Y. FRI 44398* (KEP, SAN); Chemerong FR – *Lim*, *C.L. FRI 64962* (KEP); Dungun – *Anthonysamy*, *S. SA 594* (SING); Jengai FR – *Sam*, *Y.Y. FRI 47154* (KEP, SAN), *Anon. s.n.* (SING), *s.n.* (SING), *s.n. (0105992)* (SING); Pasir Raja FR – *s.n.* (*0105989*) (SING); Rasau Kerteh FR – *Sam*, *Y.Y. FRI 47172* (KEP), *Saw*, *L.G. FRI 44992* (SING), *Lim*, *C.L. FRI 64989* (KEP, SAN), *Chan*, *Y.C. FRI 16853* (SING), *FRI 16853* (KEP), *Kochummen*, *K.M. KEP 94938* (KEP); Sg. Paka FR – *FRI 64970* (KEP); Ulu Dungun – *Yong*, *G.C. RK 3136* (KEP, SING). KEMAMAN DISTRICT: Sg. Nipah – *Corner*, *E.J.H. s.n.* (SING), *Kiew*, *R. RK 2675* (KEP, SING), *Sam*, *Y.Y. FRI 47195* (KEP), *Markandan*, *M. FRI 42698* (KEP), *Lim*, *C.L. FRI 64972* (KEP, SAN). Hulu Terengganu District: G. Padang – *FRI 67748* (KEP); Tembat FR – *Mohd. Hairul FRI 60929* (KEP), *Sam*, *Y.Y FRI 50341* (KEP). *FRI 50367* (KEP); Ulu Berang, Sg. Tersat – *Moysey*, *L. 33610* (SING).

**Notes.** This is a variable species. The typical rosette form has a woody rootstock. However, some populations on Bkt. Bauk and in Sg. Paka FR have a creeping main stem that branches and produces long, slender prostrate branches that root at lower nodes. At Sg. Paka FR, both decumbent and rosette forms are sympatric and their flowers are not different. Molecular phylogenetic results confirm that the two forms belong to the same species (Lim, 2014).

**15. *Codonoboea quinquevulnera* (Ridl.) C.L.Lim**

In Kiew & Lim, Gard. Bull. Sing. 62 (2011) 269. *Basionym: Didymocarpus quinquevulnerus* Ridl., Trans. Linn. Soc. Ser. 2, Bot.3 (1893) 328, J. Straits Branch Roy. Asiat. Soc. 44 (1905) 47, J. Asiat. Soc. Bengal, Pt. 2, Nat. Hist. 74 (1908) 758, Fl. Malay. Pen. 2 (1923) 518; Kiew, Gard.Bull. Singapore 42 (1989) 58. *Homotypic synonym: Henckelia quinquevulnera* (Ridl.) A.Weber, Beitr. Biol. Pflanzen 70 (1998) 353. *Type*: Peninsular Malaysia, Pahang, Sg. Tahan, *Ridley 2153* (lectotype K; isolectotype SING).

**Distribution.** Endemic in Peninsular Malaysia – Johor (Ulu Endau), Kelantan (Kampung La), Melaka, Pahang (Fraser’s Hill, Semangkok Pass, Tahan Valleys), Selangor (Klang Gates, Batu Tiga, Kanching FR) and Terengganu (Hulu Terengganu District).

**Ecology.** Not common, with a scattered distribution in deep shade in primary lowland forest to 200 m altitude.

**Terengganu specimens examined.** HULU TERENGGANU DISTRICT: G. Lawit – *Anthonysamy*, *S. SA 677* (KEP); Sekayu FR – *Kiew*, *B.H. s.n*. (KEP).

**16. *Codonoboea rheophytica* Kiew (see above).**

**17. *Codonoboea rugosa* (Ridl.) C.L.Lim**

In Kiew & Lim, Gard. Bull. Sing. 62 (2011) 271. *Basionym: Didymocarpus rugosa* Ridl., J. Straits Branch Roy. Asiat. Soc. 44 (1905) 45, J. Asiat. Soc. Bengal, Pt. 2, Nat. Hist. 74 (1908) 756, Fl. Malay. Pen. 2 (1923) 517. *Homotypic synonym: Henckelia rugosa* (Ridl.) A.Weber, Beitr. Biol. Pflanzen 70 (1998) 355. *Type*: Peninsular Malaysia, Kelantan, Kuala Lebir, *Gimlett s.n.* (holotype SING). *Heterotypic synonym: Didymocarpus lithophyllus* Kiew, Gard.Bull. Singapore 42 (1989) 54, Gard.Bull. Singapore 44 (1992) 38. *Type*: Peninsular Malaysia, Pahang, Sg. Tahan *Ridley 2152* (holotype K, isotype SING).

**Distribution.** Peninsular Thailand and Peninsular Malaysia – Kedah (G. Bintang), Kelantan (G. Setong), Pahang (Tahan Valley), Perak (Grik, G. Inas, Temangok) and Terengganu (Besut, Hulu Terengganu and Kemaman Districts).

**Ecology.** In the lowlands at ca. 70 m to 1000 m in Taman Negara; sometimes on shaded vertical granite rock faces.

**Terengganu specimens examined.** BESUT: Bkt Tangga, Ulu Besut – *Mohd Shah MS 4999* (KEP, SING). Hulu Terengganu District: G. Padang – *Ummul et al. FRI 64519* (KEP); Sekayu FR – *Lim C.L. et al. FRI 65235* (KEP); Tembat FR *Ong*, *P.T. et al. FRI 71359* (KEP), *Sam Y.Y. FRI 50342* (KEP), *FRI 50360* (KEP). KEMAMAN DISTRICT: Cukai – *Anthonaysam SA 590* (KEP); Sg. Nipa FR – *Chan*, *M.Y. et al. FRI 70616* (KEP).

**18. *Codonoboea salicinoides* (Kiew) C.L.Lim**

In Kiew & Lim, Gard. Bull. Sing. 62 (2011) 271. *Basionym: Didymocarpus salicinoides* Kiew, Gard. Bull. Sing. 44 (1992) 35. *Homotypic synonyms: Henckelia salicinoides* (Kiew) A.Weber, Beitr. Biol. Pflanzen 70 (1998) 355. – Paraboea salicina (Ridl.) Ridl. var. major Ridl., Fl. Malay Pen. 5 (1925) 325. *Type*: Peninsular Malaysia, Kelantan, Kuala Aring, *Yapp 193* (lectotype K; isolectotype CGE).

**Distribution.** Endemic in Peninsular Malaysia, Johor, Pahang, Kelantan and Terengganu (Besut, Dungun and Kemaman Districts).

**Ecology.** Lowlands, 20–213 m altitude, locally common on vertical earth banks, rocky stream banks or forest floor.

**Terengganu specimens examined.** besut district: Jertih, Bkt. Yong *Mohd Shah & Samsuri MS 3550* (KEP, SING). DUNGUN DISTRICT: Bkt. Bauk FR – *Anthonysamy*, *S. SA 596* (KEP), *Davison*, *G. GD 7* (SING), *GD 8* (KEP), *Jutta*, *M. FRI 59549* (KEP, SAN, SAR, SING), *Kochummen*, *K.M. FRI 2587* (KEP), *Lim*, *C.L. FRI 64954* (KEP), *Saw*, *L.G. FRI 44890* (KEP, SING); Bkt. Bandi FR – *FRI 44997* (KEP); Dungun – *Anthonysamy*, *S. SA 595* (KEP). KEMAMAN DISTRICT: Bkt. Kajang: *Anthonysamy*, *S. SA 584* (KEP), *Corner*, *E.J.H. SFN 30198* (A, K, L, LAE, SING), Sg. Nipah FR: *Kiew*, *R. RK 2654* (L, SING), *Rafidah*, *A.R. FRI 51635* (BKF, KEP, L, SAR, SING), *Sam*, *Y.Y. FRI 47238* (KEP); Ulu Bendong – *SFN 30027* (L, SING).

**19. *Codonoboea sallehuddiniana* C.L.Lim (see above)**

**20. *Codonoboea tembatensis* Kiew**

Gard. Bull. Sing. 66 (2014) 140, Fig. 2. *Type*: Peninsular Malaysia, Terengganu, Hulu Trengganu District, Tembat FR. *Kamarul*, *M. et al. FRI 67142* (holotype KEP; isotype SAN).

**Distribution.** Endemic in Peninsular Malaysia, Terengganu (Hulu Terengganu District).

**Ecology.** In lowland to hill dipterocarp forest, in valleys on shaded slopes above streams or rivers at 200–815 m elevation. It grows as individual plants or forms clumps by branching at the base.

**Terengganu specimens examined.** Hulu Terengganu District: Tembat FR – G. Tembat *Kiew*, *R. et al. FRI 57034* (KEP); Ulu Sungai Terengganu Mati – *Siti Munirah*, *M.Y. et al. FRI 67904* (KEP, K, L, SAN, SING); Sg. Pauh – *Kamarul*, *M. et al. FRI 75107* (KEP).

**Notes.** Currently both *C. norakhirrudiniana* and *C. tembatensis* are threatened by possible extinction because, from our current knowledge, they grow only in the Tembat FR area that is being clear-felled for an extension to the Kenyir Hydroelectric Dam.

## Conclusions

Botanically, the state of Terengganu is still relatively poorly known compared with the west coast flora of Peninsular Malaysia. Areas that remain largely unknown are the slopes and ridges of the mountains and the north of the state. The river systems that have been explored have proved to be biodiverse and harbour species endemic to Terengganu. The Terengganu Hills have already been identified as an area of high biodiversity importance that requires greater legal protection (Davis et al. 1995).

## Supplementary Material

XML Treatment for Codonoboea norakhirrudiniana

XML Treatment for Codonoboea rheophytica

XML Treatment for Codonoboea sallehuddiniana
